# Natural products in the management of neurodegenerative diseases

**DOI:** 10.1186/s12986-024-00800-4

**Published:** 2024-05-16

**Authors:** Rajat Goyal, Pooja Mittal, Rupesh K. Gautam, Mohammad Amjad Kamal, Asma Perveen, Vandana Garg, Athanasios Alexiou, Muhammad Saboor, Shafiul Haque, Aisha Farhana, Marios Papadakis, Ghulam Md Ashraf

**Affiliations:** 1 MM College of Pharmacy, Maharishi Markandeshwar (Deemed to Be University), Mullana-Ambala, Haryana, 133207 India; 2 Chitkara College of Pharmacy, Chitkara University, Rajpura-Punjab, India; 3 Department of Pharmacology, Indore Institute of Pharmacy, IIST Campus, Rau, Indore India; 4 Institute for Systems Genetics, Frontiers Science Center for Disease-Related Molecular Network, West China Hospital, Sichuan University, Chengdu,, China; 5 King Fahd Medical Research Center, King Abdulaziz University, Jeddah,, Saudi Arabia; 6 Department of Pharmacy, Faculty of Allied Health Sciences, Daffodil International University, Birulia, Bangladesh; 7 Enzymoics, Novel Global Community Educational Foundation, 7 Peterlee Place, Hebersham, NSW 2770 Australia; 8 Glocal School of Life Sciences, Glocal University, Uttar Pradesh, Saharanpur, India; 9 Princess Dr, Najla Bint Saud Al-Saud Center for Excellence Research in Biotechnology, King Abdulaziz University, Jeddah, Saudi Arabia; 10 Department of Pharmaceutical Sciences, Maharshi Dayanand University, Rohtak Haryana, 124001 India; 11 University Centre for Research & Development, Chandigarh University, Chandigarh-Ludhiana Highway, Mohali, Punjab India; 12 Department of Research & Development, 11741 Funogen, Athens, Greece; 13 Department of Research & Development, AFNP Med, 1030 Vienna, Austria; 14 Department of Science and Engineering, Novel Global Community Educational Foundation, Hebersham, NSW 2770 Australia; 15 Department of Medical Laboratory Sciences, University of Sharjah, College of Health Sciences, and Research Institute for Medical and Health Sciences, Sharjah, United Arab Emirates; 16 Research and Scientific Studies Unit, College of Nursing and Health Sciences, Jazan University, 45142 Jazan, Saudi Arabia; 17 Gilbert and Rose-Marie Chagoury School of Medicine, Lebanese American University, Beirut, Lebanon; 18 Centre of Medical and Bio-Allied Health Sciences Research, Ajman University, Ajman, United Arab Emirates; 19 Department of Clinical Laboratory Sciences, College of Applied Medical Sciences, Jouf University, 72388 Aljouf, Saudi Arabia; 20 Department of Surgery II, University Hospital Witten-Herdecke, University of Witten-Herdecke, Heusnerstrasse 40, 42283 Wuppertal, Germany

**Keywords:** Natural products, Neurodegenerative diseases, Neuroinflammation, Oxidative stress, Nanotechnology

## Abstract

Neurodegenerative diseases represent one of the utmost imperative well-being health issues and apprehensions due to their escalating incidence of mortality. Natural derivatives are more efficacious in various preclinical models of neurodegenerative illnesses. These natural compounds include phytoconstituents in herbs, vegetables, fruits, nuts, and marine and freshwater flora, with remarkable efficacy in mitigating neurodegeneration and enhancing cognitive abilities in preclinical models. According to the latest research, the therapeutic activity of natural substances can be increased by adding phytoconstituents in nanocarriers such as nanoparticles, nanogels, and nanostructured lipid carriers. They can enhance the stability and specificity of the bioactive compounds to a more considerable extent. Nanotechnology can also provide targeting, enhancing their specificity to the respective site of action. In light of these findings, this article discusses the biological and therapeutic potential of natural products and their bioactive derivatives to exert neuroprotective effects and some clinical studies assessing their translational potential to treat neurodegenerative disorders.

**Graphical Abstract:**

Common mechanisms, therapeutic targets, and molecular pathogenesis of neurodegeneration. It is focused on the biological and therapeutic potential of natural products and their bioactive derivatives to exert a neuroprotective effect on the pathologies of neurodegenerative diseases. 
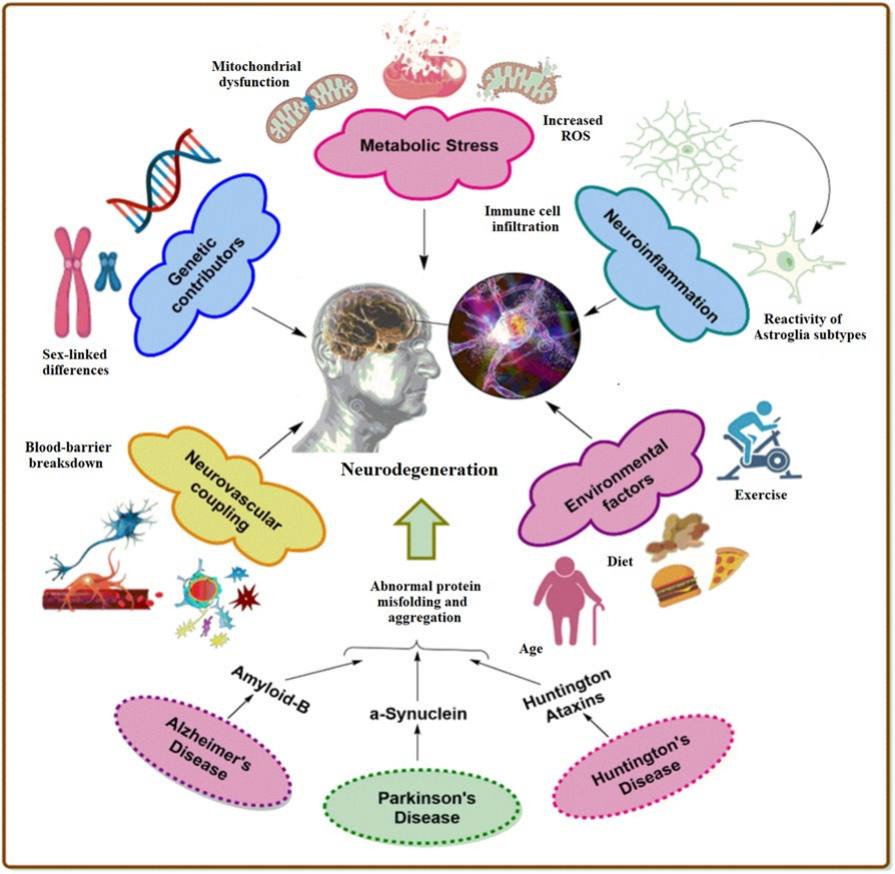

## Introduction

Neurodegenerative diseases (NDs), comprising a diverse array of disorders, are typified by the progressive degeneration of both the structural and functional components of either the central nervous system (CNS) or peripheral nervous system (PNS). Among the most prevalent of these maladies are Alzheimer's disease (AD), Parkinson's disease (PD), and spinal cord injury, which typically afflict individuals beyond the age of 60 years [57]. These debilitating conditions engender a prodigious burden on individuals and society, as the progressive loss of structural features and functions marks them. However, the root causes of several NDs remain obscure within the current healthcare system [41]. These NDs often present with a range of biological phenomena, including neuroinflammation, oxidative stress, cognitive decline, the accumulation of neurofibrillary tangles (NFTs), abnormal deposition of amyloid-β peptide (Aβ), diminution, or inadequate amalgamation of neurotransmitters and abnormal ubiquitination are linked to the progression of NDs [43]. Nevertheless, the role of aging in NDs is crucial, given their irreversible nature, the attendant social and economic burdens, and the paucity of efficacious therapeutic interventions [9].

**Fig. 1:**
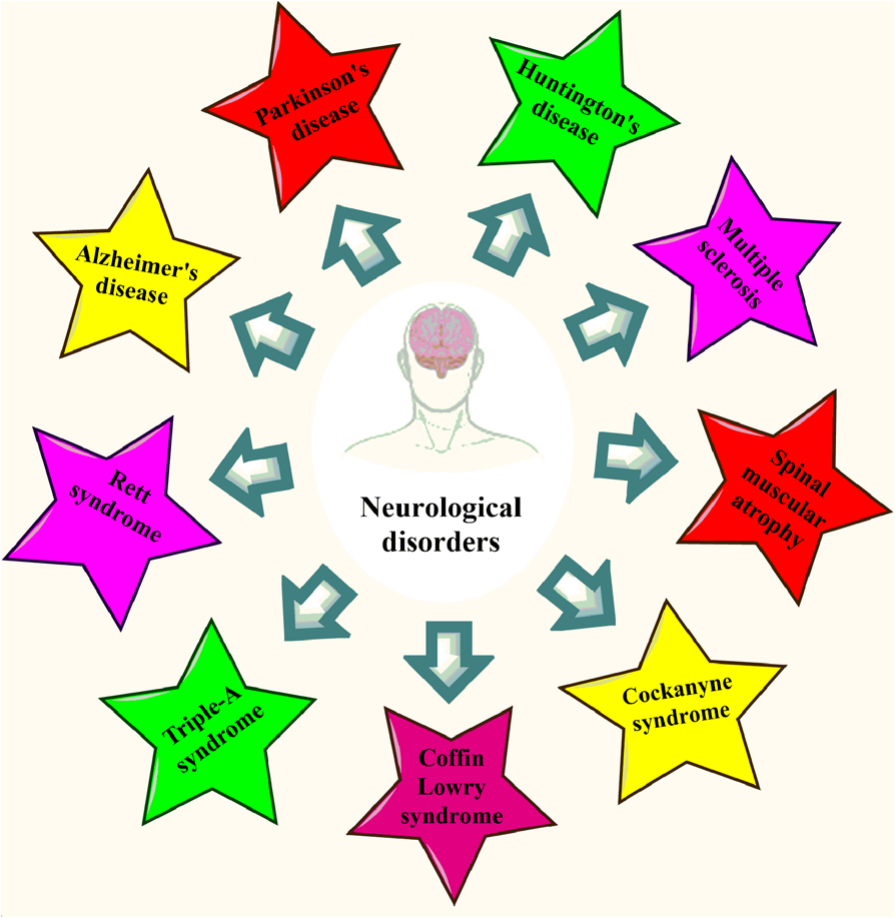
Neurological disorders

Acute neurodegeneration is a clinical condition characterized by rapid damage resulting from abrupt insult or traumatic events, i.e., strokes, traumatic brain injuries, head injuries, ischemic brain damage, subarachnoid, or cerebral hemorrhage. Conversely, chronic neurodegeneration represents a protracted ailment in which neurons undergo a neurodegenerative process that typically commences gradually and exacerbates progressively due to various aspects, ultimately causing the irreversible devastation of specific neuron populations. Chronic neurodegenerative disorders comprise a variety of conditions, including ADs, PDs, Huntington’s disease (HD), and amyotrophic lateral sclerosis (ALS) [42]. Other neurological disorders, such as spinal muscular atrophy, Cockayne's syndrome, Coffin-lowry syndrome, Triple-A syndrome, and Rett syndrome, also fall within the purview of chronic NDs [35], described in Fig. 1 and summarized in Table 1.

**Table 1:** Neurological diseases: pathogenesis, genetic basis, disease mechanism and manifestations

Neurodegenerative disease	Pathogenesis	Genetic basis	Disease mechanism	Manifestations
Multiple sclerosis	Autoimmune disorder	Multifactorial with contributions from genetic and environmental factors	Plaques/lesions formation in the spinal cord and brain, Demyelination of nerve fibers in CNS	Impaired motor function, sensory deficits, cognitive impairment
Spinal muscular atrophy	Neuromuscular disorder	Autosomal recessive inheritance with a mutation in the SMN1 gene	In the spinal cord, loss of neurons exist	Muscle weakness and atrophy, respiratory difficulties
Cockayne syndrome	Progeria syndrome	Autosomal recessive inheritance with mutations in the ERCC6 or ERCC8 gene	Impaired DNA repair mechanisms	Growth failure, premature aging, photosensitivity, neurological abnormalities
Coffin-Lowry syndrome	Intellectual disability disorder	X-linked dominant inheritance with a mutation in the RPS6KA3 gene	Defective signaling pathways in the brain	Intellectual disability, facial dysmorphism, skeletal abnormalities
Triple-A syndrome (Allgrove syndrome)	Endocrine disorder	Autosomal recessive inheritance with a mutation in the AAAS gene	Dysfunction of the adrenal gland and autonomic nervous system	Esophageal achalasia, adrenal insufficiency, lacrimal, neurological abnormalities
Rett syndrome	Neurodevelopmental disorder	X-linked dominant inheritance with MECP2 gene mutation	MECP2 gene mutation	Loss of acquired motor and language skills, intellectual disability, breathing irregularities, seizures

Alzheimer's disease (AD) is a chronic and progressive neurodegenerative disorder that predominantly distorts the human brain's vast cerebral cortex and hippocampus regions. The disease is characterized by a range of symptoms, including mental and memory impairments, cognitive decline, and personality changes, and it predominantly affects the elderly population, especially in patients above 65 years [36]. It is distinguished by two key neuropathological features i.e., (i) intracellular accretion of hyperphosphorylated tau- proteins, which form NFTs in the brain, and (ii) extracellular development and deposition of amyloid-beta (Aβ) plaques [3].

Parkinson's disease (PD) is another most common NDs that significantly impair the eminence of life and dependency and increase the menace of premature death in affected individuals [11, 16]. This disease is instigated via substantial damage to dopaminergic nigrostriatal neurons, leading to reduced motor function and induced symptoms *i.e.,* bradykinesia, resting tremor, postural imbalance, and muscular rigidity. PD are distinguished by the accretion of protein aggregates, Lewy neurites, and Lewy bodies, primarily composed of aggregated and misfolded forms of pre-synaptic protein α-synuclein [42].

Amyotrophic lateral sclerosis (ALS) is a devastating ND characterized by substantial degeneration and demise of both lower and upper motor neurons, ultimately failing the respiratory system and causing paralysis, leading to death. Despite extensive research, the underlying mechanisms of ALS remain unknown. However, various aspects, including oxidative stress, autoimmune response, impaired axonal transport, excitotoxicity, genetic factors, neurofilament aggregation, and mitochondrial dysfunction, have been considered as potential contributors to the development and progression of ALS [61]. The complex interplay of these factors leads to the liberal loss of motor neurons, ultimately leading to the debilitating symptoms of ALS.

On the other hand, Huntington’s disease (HD) is pathologically characterized by excessive dopaminergic potential and reduced functioning of gamma-aminobutyric acid (GABA) in basal ganglia and clinically characterized via cognitive deficits, atypical movements, and psychiatric disturbances [19]. This disorder is prompted by a trinucleotide repeat expansion of the CAG (nucleotide’s cytosine, adenine, and guanine) sequence in the Huntingtin (HTT) gene, that exists on the short arm of chromosome-4 [28].

## Material and methods

In this comprehensive review, we meticulously investigated the role of natural products in managing neurodegenerative diseases. Our methodology involved an exhaustive literature search, systematic data extraction, and critical analysis of relevant studies. Emphasizing transparency and rigor, we adhered to ethical guidelines to ensure the integrity of this review on natural interventions for neurodegenerative diseases.

Our search strategy encompassed databases such as PubMed and Scopus, employing keywords related to natural products and neurodegenerative diseases. Rigorous study selection involved predefined inclusion and exclusion criteria, ensuring relevance and quality. Transparent data extraction methods were applied, systematically capturing key findings to facilitate a robust analysis in our review on natural products for neurodegenerative diseases.

### Mechanism and therapeutic targets of neurodegenerative disorders

The presence of protein aggregates, oxidative stress, and inflammation within CNS marks neurodegenerative disorders. Various biological progressions have been allied to these disorders, including neurotransmitter depletion or insufficient synthesis, abnormal ubiquitination, and oxidative stress [40]. Neurodegenerative disorders are complex and multifactorial, and their underlying mechanisms are intricate. These disorders share common characteristics such as inflammation, mitochondrial deficits, abnormal cellular transport and protein deposition, excitotoxicity, intracellular Ca^2+^ overload, and unrestrained reactive oxygen species (ROS) generation. These characteristics imply the existence of converging neurodegeneration pathways, highlighting the significance of these pathways as communal markers for intervention approaches [7, 12].

Large protein aggregates within the brain, extracellular space, or neurons are among the most prominent features associated with NDs. These protein aggregates are called amyloid plaques. According to genetic evidence, one of the significant drivers of NDs is the alteration of the initially native and soluble proteins into the protein aggregates and their antecedent oligomers [26]. The common mechanisms, therapeutic targets [58], and molecular pathogenesis [41] of neurodegeneration are revealed in these articles.

### Role of naturally derived products and their metabolites in neurodegenerative diseases

Traditional medicines are crucial in fulfilling the primary healthcare requirements of developing nations, serving as a cornerstone for maintaining good health [49]. It has been reported that natural derivatives are a significant source of bioactive compounds and an imperative source of drug leads [6, 22]. In fact, according to a study, at least one-third of the drugs available in the market have their origins or were derived from different natural resources [49]. Therefore, natural derivatives continue to be extensively researched for their therapeutic potential in modern medicine. Using natural derivatives in research studies has proven to be an efficacious methodology for discovering novel, innovative, and physiologically active medicaments [47]. Natural herbs have been used to treat several ailments and improve human health and well-being for thousands of years [38].

Recently, research on natural products and their bioactive compounds as excellent therapeutic and biological agents for NDs has substantially increased. The promising potential of natural compounds in preventing and treating NDs has been widely acknowledged. However, there are some clinical concerns regarding their use, primarily due to insufficient scientific evidence supporting their efficiency and patient safety [39].

**Fig. 2:**
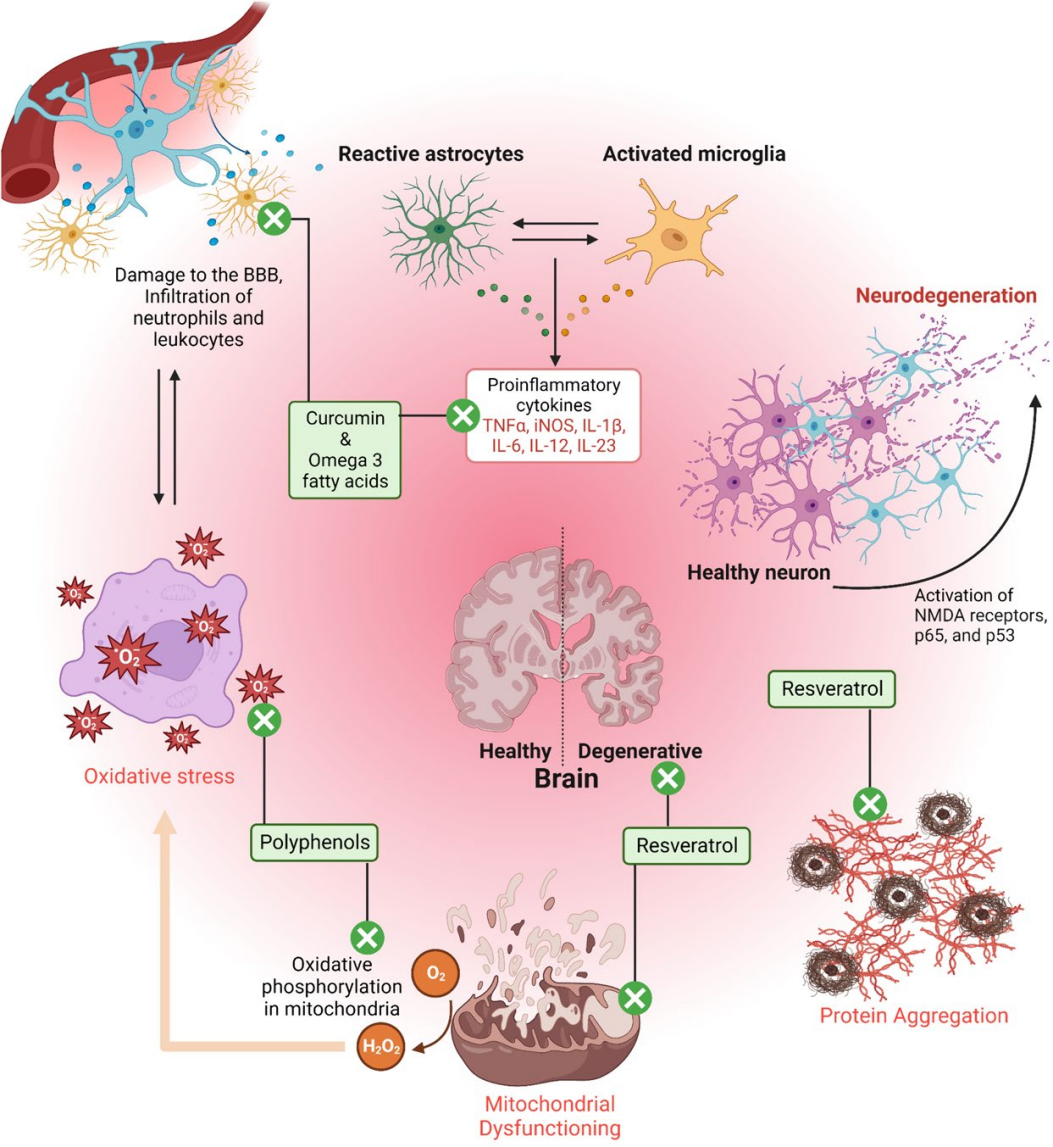
Neurodegenerative pathways and the role of bioactives in the prevention of neurodegeration

The significance of plant-based natural derivatives is evident because many of the medications currently employed to treat NDs are derived from plants. For instance, opioids alkaloids, and anticholinesterases i.e., neostigmine, physostigmine, and galantamine are derived from plants [22]. The neuroprotective characteristics of naturally derived compounds and their metabolites have been studied and reported in the literature for treating NDs. Table 2 and Fig. 2 summarizes the wide-ranging therapeutic effects of various naturally derived compounds and their metabolites in combating NDs [48, 56].

**Table 2:** Naturally derived compounds and their metabolites with neuroprotective potential in treating NDs (Pre-clinical approaches)

S. No.	Plant Source	Major phytoconstituents	Neuroprotective activities	Model Used	References
1	Blueberries (*Vaccinium angustifolium*)	Polyphenols	Reduces the ROS levels in the brain and also helps in the activation of cellular stress pathways in the brain	In vitro Neurodegenerative cell Model	[27]
2	*Capsicum annuum*	Capsicum	Prohibits the neurodegeneration in the hippocampus, cerebral cortex, and substantial nigra by diminishing the brain 5-lipoxygenase activity, subdues the intensification of nitric oxide levels and brain malondialdehyde, restores the glutathione (GSH) level, and cholinesterase activity	In vitro model /Retinone intoxication mice model	(Abdel-Salam et al. 2018) [1]
3	*Curcuma longa*	Curcuminoids (Turmeric)	Improvement in the motor functions and behavioral properties, overturns the iNOS and GFAP (Glial fibrillary acidic protein) expressions and abridges the total nitrite generation and proinflammatory cytokines in the striatum	In vitro cell model/MPTP (1-methyl-4-phenyl-1,2,3,6- tetrahydropyridine) model	(Ojha et al. 2012, Hishikawa et al. 2012) [18, 34]
4	*Dioscorea nipponica*	Diosgenin	Protects against neuroinflammation by inhibition of NF-κB, MAPK, ERK, JNK, and p38 pathways	In vitro cell line studies on RAW 264 cells	(Hirai et al. 2010) [17]
5	*Sesamum indicum*	Sesame oil	It significantly improved the learning and memory impairments, restored the elevated level of AChE and Aβ overexpression, and mitigate the oxidative stress status in the brain	In vitro Rat model of AD	(Mohamed et al. 2021) [32]
6	*Vitis vinifera*	Reserveratrol	Inhibits the amalgamation and liberation of pro-inflammatory mediators, constraints iNOS, NF-κB, COX-II, and AP-1, and promotion of IL-10	In vitro cell line studies/ 3-(4,5-dimethyl-thiazol-2-yl)-2,5-diphenyl tetrasodium bromide (MTT) assay in BV2 microglia cells	(Song et al. 2014) [51]
7	*Nicotiana tabacum*	Osmotin	Reduction of Aβ accretion and expression of BACE-1, ameliorates memory impairment, prevents Aβ-induced neurotoxic effects of neuronal- HT22 cells, and reverses synaptic deficits	In vitro Y Maze test	(Ali et al. 2015) [2]
8	*Coptis chinensis*	Berberine	Triggers the regulations of AKT/GSK-3β/Nrf2, persuades the secretion of NGF and BDNF, and inhibition of COX-II, iNOS, TNF-α, NF-κB, and IL-1β	In vitro cell line studies/MTT assay in BV2 microglia cells	(Lee et al. 2012; Jia et al. 2012) [20, 23]
9	*Morus alba*	Quercetin	Inhibits COX-II, GSK-3β, 5-LOX enzymes, and NF-κB activation, and intricates in the free radical scavenging	In vitro animal model/MTTP (1,2,3,6-tetrahydropyridine) induced neurodegeneration	(Pany et al. 2014) [37]
10	*Vitis vinifera*	Polyphenols	Abridges iNOS, PARP, and TNF-α expression and level of nitro-tyrosine, and subdues Bcl-2 and caspase-3 expressions	Mice model of autoimmune encephalomyelitis	(Giacoppo et al. 2015) [14]
11	*Zingiber officinale*	6-shogaol (Ginger)	Persuades the secretion of NGF, BDNF, and GDNF, inhibition of iNOS, IL-1β, TNF-α, p38, NF-κB, Bax, PGE2, NO, and ROS, and upsurges Bcl-2 levels	Primary Cell culture	(Ha et al. 2012) [15]
12	*Ginkgo biloba*	Ginkgolide B	Overwhelms PI3K/Akt and NF-κB pathways, upregulation of expression of anti-apoptotic proteins, and reduces LDH, ROS and caspase3	Primary Cell culture/ MTT assay	(Nabavi et al. 2015; Xiao et al. 2010) [8, 62]
13	*Panax ginseng*	Ginsenoside Rg3	Triggers the cAMP/MAPK and Trk-mediated neurogenesis, inhibition of NF-κB, TNF-α, iNOS, and IL-1β	MTT Assay with BV2 microglial cell lines	(Joo et al. 2008) [21]

### Clinical studies on the translational prospectives of natural derivatives to treat neurogenerative disorders

Clinical trials are currently underway to develop and test a wide range of interventions for NDs. These interventions encompass a broad spectrum of therapeutic approaches, including cognitive enhancement, anti-amyloid and anti-tau interventions, anti-neuroinflammation interventions, neuroprotection, and neurotransmitter modification, in relieving behavioral psychological symptoms. A range of natural compounds have shown promise in clinical trials, and ongoing investigations are focused on elucidating their mechanisms of action and potential therapeutic benefits, as depicted in Table 3. Table 4 further details clinical trials and human evaluation doses for various phytochemicals demonstrating neuroprotective effects.

**Table 3:** Clinical trials on phytochemicals/phytoconstituents employed in the management of NDs

S.No.	Phytoconstituents	Mechanism of action	NCT number	Sponsor	Status
1	Ginkgo biloba	Antioxidant activity and anti-amyloid aggregation	NCT03090516	Nanjing Medical University	Recruiting
2	Guanfacine	Alpha-2A-adrenoceptor agonist, an effective 5-HT2B receptor agonist	NCT03116126	Imperial College London	Recruiting
3	Coconut oil	Attenuation in the expression of ADP-ribosylation factor-1 protein	NCT01883648	University of South Florida	Terminated
4	Caffeine	Antagonizes the adenosine receptors and improves the motor system and also has an impact on Levodopa serum concentrations	NCT01738178	Research Institute of the McGill University Health Centre	Recruitment- Completed
5	Huperzine-A	Cholinesterase inhibitor, also decreases the levels of soluble and insoluble beta-amylase levels in AD	NCT00083590	-	Recruitment- Completed

**Table 4:** Clinical trials and human evaluation doses for various phytochemicals as neuroprotective

S. No.	Phytochemicals	Mechanism of action	Dose	Clinical Trial Data	References
1	Edaravone (Trade Name: Radicava)	Free radical scavenger	3 mg/kg, (two times a day for 14 days)	The compound showed great potential in the management of ischemic stroke and now has been sanctioned for the treatment of the same in Japan in 2001	(Watanabe et al. 2018) [59]
2	Dl-3-n-butylphthalide	A multi-target drug exerts its actions via antioxidant, anti-apoptosis, and anti-inflammation, and also protects the mitochondria	40 mg/kg-200 mg/kg	The compound was found to be effective in the management of Ischemic stroke and was sanctioned for the treatment of the same in China in 2002	(Liao et al. 2018) [25]
3	Baicailein from *Scutellaria baicalensis*	A multi-target drug exerts its actions via antioxidants, anti-apoptosis, and inflammation and also protects the mitochondria. Also inhibits LOX/p38/cPLA2 pathway, and overwhelms the NF-κB activation	24 mg/kg (i.v) dose	Phase I clinical trials, single-center, randomized, placebo-controlled, double-blind, single dose-escalation, healthy male and females volunteers were used	(Li et al. 2014) [24]
4	Scutellarin (scutellarein-7-O-glucuronide) from *Erigeron breviscapus*	Acts by suppressing microglial activation and inflammation	30–40 ml/day for 8–12 days was found to be safe and effective along with Dengzhanxixin	Great potential for its clinical use. Recently, an injection of Dengzhanxixin is approved for the management of ischemic shock (approval number Z53021569) in China. Scutellarin is the main component present in this injection	(Wang et al. 2018) [55]
5	Naringenin	It inhibits NF-κB, lessers the inflammation, decreases the BBB dysfunction, and enhances Nrf2-mediated anti-oxidation	120 (mg/kg) i.v for 15 min	Phase 1 clinical trials NCT0358255, recruiting	(Nouri et al. 2019) [33]

### A glimpse of recent patents granted or filed on phytoconstituents for their neuroprotective action

The brain is undoubtedly one of the most sensitive and crucial organs in the human body, and any damage inflicted upon it can have catastrophic consequences. However, recent research investigations have revealed that numerous phytoconstituents hold promise in reversing brain damage and preventing further harm. Several compounds have been studied extensively for their neuroprotective actions in the past years, with many receiving patents. The potential of these phytoconstituents lies in their ability to mitigate the damage triggered by inflammation, oxidative stress, and other factors contributing to neurodegeneration. By protecting and repairing damaged neurons and improving overall brain function, these compounds offer a novel and promising approach to treating a wide range of NDs. Furthermore, their use could help to discourse the unmet medical needs in this field, which have remained largely unfulfilled due to the limited effectiveness and significant side effects associated with existing treatments. In this way, the discovery of neuroprotective phytoconstituents represents a significant breakthrough in neurology and holds enormous promise for improving the eminence of life of those affected by NDs.

Table 5 represents the data of patents of neuroprotective agents along with their therapeutic receptors.

**Table 5:** Patented data of various neuroprotective phytoconstituents

S. No.	Name of the compound	Therapeutic action	Details of Patent
1	*Rhizoma coptidis* (*Coptis chinensis*, *Radix scutellariae*, *Cortex phellodendri* in 3:2:2:3 dry weight	Used in the treatment of stroke, ADs, and dementia	US patent No US9375457B2
2	Cannabinoids such as cannabidivarin, cannabichromene, and cannabidivarin acid	Used and approved for the treatment of ADs	US patent No US10258580B2
3	Limonoids	Used for the prophylaxis and treatment of neurodegeneration	US patent No US9289412B2
4	Elazi tannins	Used for the treatment of delirium, dementia, learning, and attention deficit disorder (ADD)	Japanese Patent No JP6935331B2
5	Novobiocin analogs	Used for the treatment of beta-amyloid disorder, and is most preferably ADs	US patent No US7960353B2
6	Cardiac glycosides	Used for the treatment of ADs, HDs, or stroke	Australia Patent No AU2016262784B2

### Role of nanotechnology in the drug formulation and development of phytochemicals

Phytoconstituents display various therapeutic functions, including anticancer, antioxidant, and neuroprotection properties. However, their efficacies are often limited by issues related to solubility and bioavailability. The scale-up issue from laboratory to commercialization has hindered the application of natural compounds in the pharmaceutical industry, primarily due to solubility and bioavailability concerns when administered in conventional forms. To overcome these limitations, nanotechnology has emerged as a potential solution. Specifically, nanosponges, nanoemulsions, nanogels, nano micelles, and nanoparticles are innovative drug delivery systems based on nanotechnology that can improve the solubility and specificity of naturally derived bioactive compounds [31, 44].

Nanotechnology-based drug delivery methods can potentially enhance the specificity of natural bioactive compounds by precisely targeting their site of action. This targeted approach can effectively prevent receptor-specific diseases, such as breast cancer, by targeting HER receptors with increased efficacy. Furthermore, researchers are currently investigating brain targeting and the target of neurological receptors for diagnosing, preventing, and managing NDs. Thus, the application of nanotechnology-based drug delivery can facilitate the utilization of neuroprotective phytoconstituents in the pharmaceutical industry and potentially revolutionize the treatment of various diseases. Indeed, one of the most significant advantages of using nanotechnology-based drug delivery systems is the ability to minimize the side effects of various drugs. This is primarily due to the improved bioavailability, which reduces both the dose and dose-related toxicity. With the help of nanotechnology, drugs can be delivered more precisely to the envisioned site of action, and diminishing their effects on healthy tissues. Additionally, the use of nanocarriers can help to protect drugs from premature degradation or elimination, further enhancing their therapeutic potential [4, 5, 62].

Nanotechnology has proven to be a favorable solution in enhancing the efficacy of herbal compounds. Although synthetic and semisynthetic compounds also face the issue of limited bioavailability, their problems are related to poor solubility, efficacy, and bioavailability, which can be resolved by creating salt forms or derivatives. However, this is not a feasible solution for herbal compounds. Nanotechnology, on the other hand, offers a more effective approach to addressing these issues. Nanotechnology offers several methods to enhance the efficacy of poorly bioavailable compounds. The bioavailability of the compounds can be improved by formulating nanoformulations such as solid lipid nanoparticles, nanocrystals, and nanosponges. These nanoformulations prevent their first-pass metabolism and degradation of these compounds while aiding in their targeting of specific sites of action. As a result, the bioavailability and efficacy of the drugs can be enhanced significantly.

**Fig. 3:**
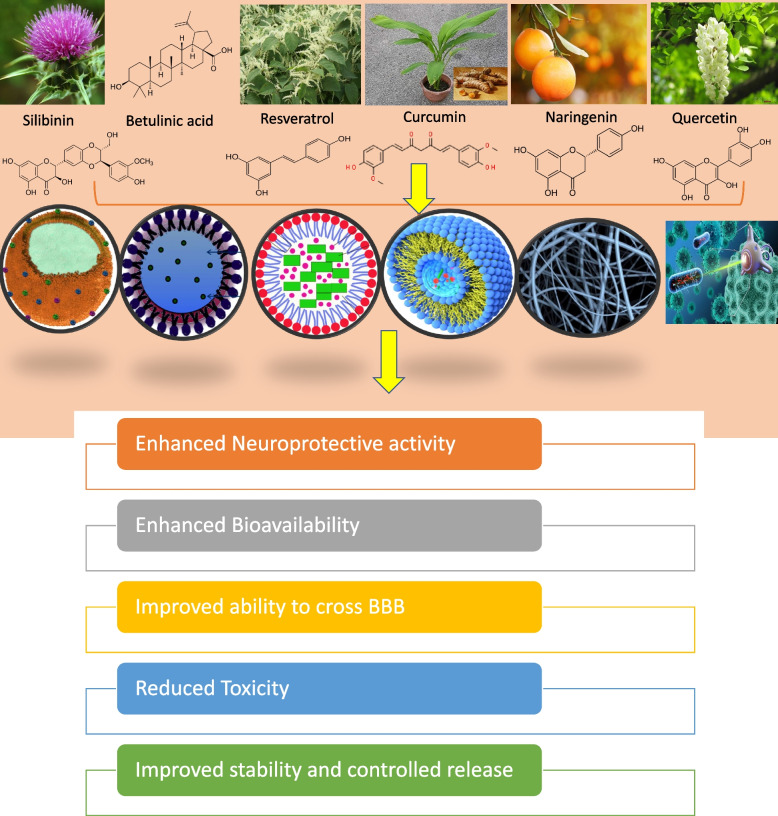
Few phytoconstituents and various nanoformulations are used nowadays

Furthermore, using lipids as drug carriers has emerged as a promising area of research. Lipids can protect fragile drugs from degradation, and incorporating these components into lipid carriers can facilitate safe delivery to their targets while preventing metabolic degradation. Another approach for enhancing efficacy is hydrogels, which can stabilize the bioactivity and improve their delivery to specific targets. These strategies offer great potential for developing more effective and efficient treatments for various diseases. Some of the nanotechnology-based delivery methods for phytoconstituents are mentioned in Table 6 and Fig. 3. These mentioned compounds were selected based on their neuroprotective data available on various data bases [29, 46].

**Table 6:** Nanotechnology-based phytochemicals used for the treatment of NDs

Sr. No.	Phytoconstituents	Drug Delivery System	Combating disease	References
1	Resveratrol	Nanostructured lipid carriers, and solid lipid nanoparticles (SLNPs)	Treatment of ADs	(Fonseca-Santos et al. 2015) [13]
2	Curcumin	PLGA based nanoparticles	Treatment of ADs	(Yavarpour-Bali et al. 2019) [63]
3	Naringenin	Nanoemulsions	To combat PDs and treatment of ADs	(Nouri et al. 2019) [33]
4	Quercetin	PLGA nanoparticles, nanoencapsulation	To combat PDs and treatment of ADs	(Enteshari Najafabadi et al. 2018) [10]
5	Epigallocatechin-3 gallate	Selenium nanoparticles coated with Tet-1 peptide	Increase neuronal alpha-secretase, Increased oral bioavailability	(Singh et al. 2015) [50]
6	Ferulic acid	SLPNs	Antioxidant action	[52]
7	Huperzine-A	Lactoferrin-conjugated N-trimethylated chitosan nanoparticles	Increased mucoadhesion	(Wen et al. 2017) [60]

### Challenges, limitations and endorsements for future research

Natural herbs, either in their whole form or as extracts, are widely recognized for their potential neuroprotective properties against various NDs. Although numerous preclinical investigations have established the efficiency of these herbs for treating neurodegeneration, there has been a significant gap in successfully translating these findings from research to commercialization. While preclinical evidence is abundant, clinical testing remains limited. As a result, the potential of natural herbs as a viable treatment option for neurodegenerative disease remains largely unexplored. Using natural products for neuroprotection faces various challenges related to their physicochemical stability, solubility, metabolism, crossing the blood–brain barrier (BBB), and therapeutic efficacy. Even though several natural substances, such as Resveratrol, turmeric, and apigenin, have been shown to possess multiple neuroprotective properties, their efficacy is hampered by poor stability, solubility, and bioavailability.

Addressing the complexities of natural products in neurodegenerative disease management, challenges include the need for standardized methodologies, rigorous clinical trials, and understanding intricate molecular mechanisms. Limitations encompass variability in bioavailability and inconsistent study designs. Future research should prioritize large-scale, well-controlled trials, exploring synergistic effects of natural compounds. Endorsements for advanced technologies, such as omics approaches, could unravel novel therapeutic targets. Additionally, interdisciplinary collaboration between researchers, clinicians, and industry partners is essential for advancing the field. Overcoming these challenges and embracing innovative strategies will pave the way for more efficacious natural product-based interventions in neurodegenerative disease treatment [30, 45].

Furthermore, the BBB poses a significant obstacle for these substances, preventing them from crossing the bloodstream to the brain. However, nanotechnology and nanocarriers have the potential to improve their solubility, bioavailability, and stability. The use of encased nanocarriers to deliver natural compounds has shown significant improvements in their bioavailability and stability. Several types of nanocarriers, such as nanosuspension, nano gels, nano micelles, and nanostructured lipid carriers, have been formulated to deliver phytoconstituents. These nanocarriers help in the phytoconstituents entrapment and considerably improve their stability, as demonstrated by recent research [39, 44, 53, 54].

## Conclusion

Preclinical studies have provided compelling evidence of the therapeutic potential of phytoconstituents as neuroprotectors. The documented bioactivities of natural substances, such as scavenging of reactive oxygen species, antioxidant action, antiproliferative activity, and antibacterial and anticancer properties, along with their neuroprotective effects, are well established. Several natural substances, including luteolin, hesperidin, resveratrol, and genistein have demonstrated efficacy against neurodegeneration. However, their therapeutic potential is limited by solubility, stability, and efficacy issues that impede their clinical translation. Recent studies have shown that natural substances can be made more therapeutically effective by incorporating them into nanocarriers, such as nanogels, nanoparticles, and nanostructured lipid carriers. This strategy can potentially overcome natural substances' limitations and significantly improve bioactive compounds' stability, solubility, and specificity, thereby enhancing their therapeutic activity.

## Data Availability

All the available data are included in the manuscript. No new data was generated.

## Abbreviations

5-HT2B: 5-Hydroxytryptamine receptor 2B
5-LOX: Arachidonate 5-lipoxygenase
AAAS: Achalasia–Addisonianism–Alacrima syndrome
Aβ: Amyloid-β peptide
AChE: Acetylcholine
AD: Alzheimer's disease
AKT: Ak strain transforming
ALS: Amyotrophic lateral sclerosis
AP-1: Activating protein-1
BACE-1: Beta-site amyloid precursor protein cleaving enzyme 1
BBB: Blood-brain barrier
Bcl-2: B-cell lymphoma 2
BDNF: Brain-derived neurotrophic factor
CAG: Nucleotide’s cytosine, adenine, and guanine
cAMP: Cyclic adenosine monophosphate.
CNS: Central nervous system
COX-II: Cyclooxygenase II
cPLA2: Cytosolic phospholipases A_2_
ERCC6: ERCC Excision Repair 6, Chromatin Remodeling Factor
ERCC8: ERCC excision repair 8, CSA ubiquitin ligase complex subunit
ERK: Extracellular signal-regulated kinase
GABA: Gamma-aminobutyric acid
GDNF: Glial cell line-derived neurotrophic factor
GFAP: Glial fibrillary acidic protein
GSH: Glutathione level
GSK-3β: Glycogen synthase kinase-3 beta
HD: Huntington’s disease
HT22: Immortalized mouse hippocampal neuronal cell line
HTT: Huntingtin gene
iNOS: Inducible nitric oxide synthase
JNK: Jun N-terminal kinase
IL-10: Interleukin 10
IL-1β: Interleukin-1 beta
LDH: Lactate dehydrogenase
MAPK: Mitogen-activated protein kinase
MECP2: Methyl-CpG Binding Protein 2
MTT: 3-[4,5-Dimethylthiazol-2-yl]-2,5 diphenyl tetrazolium bromide assay
NDs: Neurodegenerative diseases
NF-κB: Nuclear factor kappa-light-chain-enhancer of activated B cells
NFTs: Neurofibrillary tangles
NGF: Nerve growth factor
NO: Nitric oxide
Nrf2: Nuclear factor erythroid 2–related factor 2
PARP: Poly (ADP-ribose) polymerases
PD: Parkinson's disease
PGE2: Prostaglandin E2
PI3K: Phosphoinositide 3-kinase
PNS: Peripheral nervous system
ROS: Reactive oxygen species
RPS6KA3: Ribosomal Protein S6 Kinase A3
SLNPs: Solid lipid nanoparticles
SMN1: Survival of motor neuron 1
TNF-α: Tumor necrosis factor alpha
